# Role for Growth Regulation by Estrogen in Breast Cancer 1 (GREB1) in Hormone-Dependent Cancers

**DOI:** 10.3390/ijms19092543

**Published:** 2018-08-28

**Authors:** Meng Cheng, Stephanie Michalski, Ramakrishna Kommagani

**Affiliations:** Center for Reproductive Health Sciences, Department of Obstetrics and Gynecology, Washington University School of Medicine, St. Louis, MO 63110, USA; meng.cheng@wustl.edu (M.C.); saemichalski@gmail.com (S.M.)

**Keywords:** GREB1, steroid hormone, estrogen, progesterone, androgen, breast cancer, ovarian cancer, prostate cancer

## Abstract

Sex hormones play important roles in the onset and progression of several cancers, such as breast, ovarian, and prostate cancer. Although drugs targeting sex hormone function are useful in treating cancer, tumors often develop resistance. Thus, we need to define the downstream effectors of sex hormones in order to develop new treatment strategies for these cancers. Recent studies unearthed one potential mediator of steroid hormone action in tumors: growth regulation by estrogen in breast cancer 1 (GREB1). *GREB1* is an early estrogen-responsive gene, and its expression is correlated with estrogen levels in breast cancer patients. Additionally, GREB1 responds to androgen in prostate cancer cells, and can stimulate the proliferation of breast, ovarian, and prostate cancer cells. Recent studies have shown that GREB1 also responds to progesterone in human endometrial cells, suggesting that *GREB1* is a pan steroid-responsive gene. This mini-review examines evidence that GREB1 participates in several hormone-dependent cancers and could be targeted to treat these cancers.

## 1. Hormone-Dependent Cancers

Steroid hormones are a selective branch of signaling molecules, which are biosynthesized from cholesterol derivatives in the mitochondria [1]. Steroid hormones are broadly classified into two subcategories based on their biosynthesis: corticosteroids and sex steroids [2]. Produced in the adrenal cortex, corticosteroids regulate a wide array of physiological processes, including immune, inflammatory, and stress responses. Notable corticosteroids include the glucocorticoid, hydrocortisone, mineralocorticoid, and aldosterone hormones. Produced in the gonads, hormones in the sex steroid class (i.e., estrogen, progesterone, and androgen) are vital for the development and maintenance of sexual characteristics as well as for general reproductive function [2]. Despite their role in normal reproductive development, sex steroids have also co-opted to play a role in the development and progression of several human cancers. 

Sex-steroid hormones exert their physiologic and pathological function through their cognate receptors (steroid receptors), including estrogen receptors (ERs), androgen receptors (ARs), and progesterone receptors (PRs). All of these receptors belong to the nuclear receptor superfamily and consist of tissue-specific subtypes [3,4]. For example, produced differentially in various tissues, two distinct receptors, ERα and ERβ, exert distinct physiological and pathophysiological functions. ERα is the predominant receptor in the uterus, breasts, and ovaries, whereas ERβ plays significant roles in the bone, kidney, and cardiovascular systems [5]. Importantly, numerous findings revealed a key role for these hormones and their cognate receptors in human cancer onset and progression [4,6,7]. For example, the majority of breast cancer cell lines express the estrogen receptor [8,9,10], and the first-line therapy Tamoxifen selectively antagonizes ER [11,12]. Likewise, the risk of epithelial ovarian cancer is increased by long-term exposure to estrogen, as occurs in early menarche, late menopause, and in the context of hormone replacement therapy [13,14]. Indeed, one study of 394 serous epithelial ovarian cancer patients reported that 86% of tumors were ER-positive [15]. Moreover, when ER-positive epithelial ovarian cancer cells were implanted in the peritoneal cavity of mice, estrogen significantly increased tumorigenesis, promoted lymph node metastasis, and decreased survival [16,17]. Additionally, prostate cancer initiation and progression is regulated by androgen [18]. Thus, common treatment options for prostate cancer include orchiectomy and hormonal therapy, both of which function by restricting the androgen/androgen receptor interaction [4]. 

Although anti-hormonal therapeutics have been used to treat these hormone-dependent cancers, such drugs are often ineffective in the long term as the cancers develop drug resistance. For example, Tamoxifen, a selective estrogen receptor modulator (SERM), is widely used in breast cancer therapy [11]. However, about one-third of patients will acquire drug resistance and suffer from tumor recurrence [19]. Overcoming this challenge will require defining the mechanisms by which hormones drive these cancers and identifying new therapeutic targets.

## 2. Nuclear Receptor Coactivators in Hormone-Dependent Cancers

**Figure 1:**
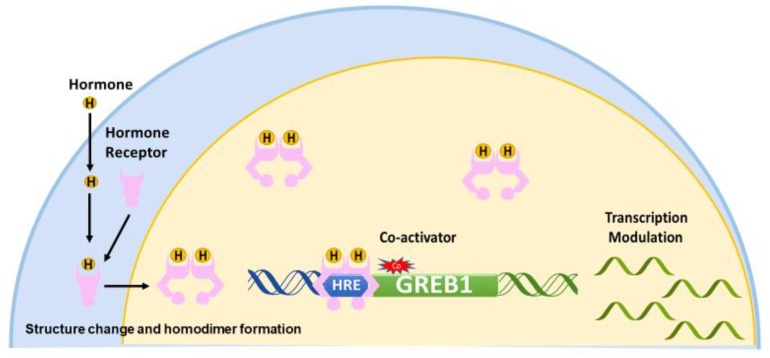
Model of the classic hormone-signaling pathway. The hormone enters the cell andbinds to a hormone receptor (HR). The arrows represent steps involved in activation of HR by its hormone. The HR forms a homodimer, undergoes a structural change to expose the hormone response element (HRE) binding domain, and enters the nucleus. Then, in conjunction with coactivators, HR binds an HRE to modulate the transcription of downstream genes such as GREB1. The arrows represent steps involved in activation of HR by its hormone.

Nuclear receptors, including ER, AR, and PR, share a common mechanism of regulation. Upon the binding of a hormone to its cognate receptors, the receptor undergoes a conformational change to expose the hormone response element binding site and form a homodimer [5]. Upon interaction of the receptor with a coactivator, the homodimer binds to hormone response elements and modulates the transcription of hormone-dependent genes [3,20] (Figure 1). 

The steroid receptor coactivator (SRC) family was the first identified coactivator class for nuclear receptors [21]. Three members, SRC-1, SRC-2, and SRC-3, are part of this key coactivator family. All three isoforms share conserved domains, including the N-terminal basic helix-loop-helix-Per/ARNT/Sim (b-HLH-PAS), the central nuclear receptor interacting domain, and two C-terminal activation domains, AD1 and AD2 [22]. The N-terminal b-HLH-PAS domain is highly conserved in the SRC family. The central nuclear receptor interacting domain contains three Leu-X-X-Leu-Leu (LXXLL) motifs, which are crucial for the coactivator’s function toward nuclear receptors. The X-ray crystal structure shows that LXXLL motifs have a two-turn α-helical conformation. The three conserved leucines align on the face of the helix and fit into the hydrophobic channels of the ligand-binding domain (LBD) on nuclear receptors [23]. The C-terminal activation domain can recruit different coactivators to modulate the transcription [22,24]. 

Recent advancements revealed an indispensable role for SRCs in steroid hormone-dependent functions in hormone-dependent carcinogenesis. In hormone-dependent cancers, all three members of the SRC family are expressed and are associated with prognosis [25,26,27]. In addition, SRC-1 and SRC-3 can stimulate cancer cell proliferation and promote cancer metastasis [28,29]. The expression of SRC-1 in breast cancer patients is associated with acquired drug resistance and relapse after endocrine therapy [30]. Likewise, in ovarian cancer, SRC-3 is elevated in high-grade ovarian epithelial cancer tissue [31]. SRC-3 considered as a poor prognostic factor and confers platinum resistance in ovarian epithelial cancer [32]. In endometrial cancers, the high expression of *SRC-3* mRNA correlates with the advanced stage of cancer and poor prognosis [33]. Recent advancements highlighted a central role for SRC members in prostate cancer cells [34]. For example, all three family members were found to be essential for cancer cell survival and promote metastasis [35,36,37]. In contrast, the inhibition of the expression of SRC-3 can improve the sensitivity of human breast cancer cell line MDA-MB-231 to histone deacetylase inhibitors [38]. Further, the downregulation of SRC-3 reduces ovarian cancer cell migration and metastasis [31]. These aforementioned findings highlight an indispensable role for SRCs in hormone-dependent cancer onset and progression. 

Interestingly, SRC-3 is reported to interact with growth regulation by estrogen in breast cancer 1 (GREB1) and increase estrogen-induced transcription in breast cancer cell line MCF-7 cells [39]. We found that *GREB1* is transcriptionally activated by SRC-2 via the progesterone–PR axis in endometrium [40], and a novel fusion gene, *GREB1-SRC-2*, was detected in uterine sarcoma [41]. These evidences point out that GREB1 may play a vital role in hormone-dependent cancers. In this review, we will argue that GREB1 is a good candidate for development of therapeutics for breast, ovarian, prostate, and perhaps even uterine and testicular cancers. 

## 3. GREB1 Structure and Function

**Figure 2:**
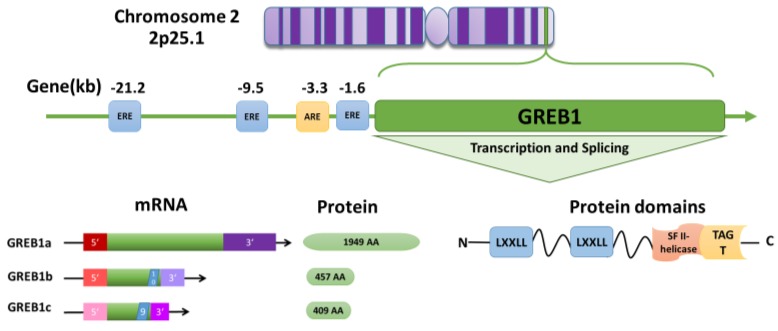
Model of growth regulation by estrogen in breast cancer 1 (GREB1) structure and transcription. The human *GREB1* gene is located on 2p25.1. Three upstream estrogen response elements (EREs) located at −21.2, −9.5, and −1.6 kb and one androgen response element (ARE) located at −3.3 kb can modulate GREB1 transcription. The GREB1 gene produces at least three isoforms: GREB1a (NM_014668.3 → NP_055483.2), GREB1b (NM_033090.2 → NP_149081.1), and GREB1c (NM_148903.2 → NP_683701.2), which have alternate 5′ and 3′ UTRs. Splicing after exons 10 and 9 results in short GREB1b and GREB1c isoforms, respectively. The GREB1a protein contains two LXXLL motifs and a superfamily II (SFII) helicase module fused with a glycosyltransferase (TAGT) motif in the C-terminus.

*GREB1* was first discovered in brain tissue [42] and later identified as an early estrogen-responsive gene in breast cancer [43]. The *GREB1* gene is highly conserved across species and is expressed in many tissues, including the brain [42], mammary gland [43], ovary [16], prostate [44], and endometrium [40]. In humans, the *GREB1* gene is located at 2p25.1, spans 108.68 kb, and contains 38 exons and 40 introns [43,45] (Figure 2). *GREB1* is expressed as three distinct isoforms: GREB1a, GREB1b, and GREB1c [43]. GREB1a is the full-length isoform, whereas variant splicing and the introduction of early termination codons result in GREB1b and GREB1c, which terminate after exons 10 and 9, respectively [46] (Figure 2). 

Very little is known on the functional differences between these three isoforms. Much of our understanding of the GREB1 function comes from the analysis of its protein domains. At the C-terminus, GREB1 contains a circularly permuted superfamily II (SFII) helicase fused to a ten-eleven translocation/J binding protein (TET/JBP) and glycosyltransferase (TAGT) domain [47] (Figure 2). The SFII helicase is presumed to bind nucleic acids, but has no catalytic activity [47], and TAGTs can glycosylate pyrimidines in DNA [47]. The glycosylation is a key modification observed in different types of cancers, including breast, ovarian, and prostate cancers [48]. Glycosylation facilitates the cell adhesion, proliferation, and metastasis of breast cancer [49]. Six glycosylated genes—FUT1, FUCA1, POFUT1, MAN1A1, RPN1 and DPM1—are reported to be associated with the prognosis of breast cancer [50]. Further, glycosyltransferase gene expression is correlated with the prognosis of ER-positive breast cancer [51]. However, the importance of GREB1-mediated DNA modification is unknown. At the N-terminus, GREB1 contains two LXXLL motifs, which are commonly found in coactivators and mediate interactions with nuclear receptors [52,53] (Figure 2). However, the LXXLL motifs are not necessary for GREB1 to interact with ER [46], and although all three GREB1 isoforms bind to ER [53], they may not function as coactivators. Instead, exogenously expressed GREB1a can independently enhance the expression of ER target genes PS2 and SDF1, while exogenously expressed GREB1b had no effect [46]. These findings suggest that the GREB1 isoforms might have differential effects on ER driven gene expression.

## 4. Hormone Regulation of GREB1 

The classic hormone-signaling pathway begins with a hormone binding to its cognate hormone receptor, causing the receptor to homodimerize [5] and undergo a structural change to expose the hormone response element binding site [54]. While the hormone-binding domain is located at the C-terminus, the hormone response element binding domain is in the central region of the receptor. The hormone-bound homodimer enters the nucleus where, in conjunction with a coactivator, it binds to hormone response elements in the upstream of GREB1 and then modulates the transcription of target genes [3,20] (Figure 1). 

Several lines of evidence indicate that *GREB1* is regulated by the hormones estrogen, progesterone, and androgen [54,55,56,57,58]. First, *GREB1* expression was markedly elevated in the mammary tissue of female cynomolgus macaques during the pubertal stages, when estrogen levels were high, and decreased after menopause, when estrogen levels were low [56]. Second, in breast cancer patients, *GREB1* expression correlated with estrogen levels [57]. Third, throughout women’s reproductive years, *GREB1* expression in the endometrium fluctuates in accordance with estrogen levels [59]. Fourth, *GREB1* expression increased during progesterone-mediated endometrial stromal cell decidualization, and progesterone receptor knockdown led to a decrease in GREB1 transcript levels [40]. Further, three estrogen response elements (EREs) [54,58] and at least one androgen response element (ARE) [44,53] are located upstream of the GREB1 transcription start site (Figure 2). Consistent with this, the expression of GREB1 correlated with the expression of hormone receptors in reproductive tissues in human (Table 1). The known functions of GREB1 in reproductive cancers will be detailed in the following sections of this review. 

## 5. Role of GREB1 in Breast Cancer

Estrogen and progesterone play a vital role in mammary gland growth and cancer development [60]. Breast cancer is the most prevalent form of cancer in women [61]. In the mammary gland, ERα is the predominant subclass of ER, and is expressed in the majority of breast cancer cells. Estrogen is a key mediator in the development and progression of breast cancer [7], and several studies indicate that ER regulates *GREB1* expression. Cancer cells expressing the ER are classified as ER-positive breast cancers [8]. In ER-positive breast cancers, the plasma level of estradiol and the expression of ERs are positively correlated with *GREB1* expression [62]. The younger (age ≤ 45 years) group of breast cancer patients who had higher estrogen levels showed significantly higher *GREB1* expression than the older (age ≥ 70 years) group [63]. Additionally, the majority of breast cancer cell lines, including MCF-7, T-47D, and BT-474 [9,10], are ER-positive and express *GREB1* at high levels. Conversely, ER-negative breast cancer cell lines MDA-MB 231 and SUM 225 express GREB1 at lower levels [64]. GREB1 appears to help regulate the transcription of ER target genes, as the chromatin immunoprecipitation (ChIP) sequencing analysis of MCF-7 cells revealed that approximately 95% of the GREB1-bound regions were also bound by ER [53]. Upon GREB1 knockdown in MCF-7 cells, nearly half of the estrogen-responsive genes were no longer differentially expressed, and the cells were less able to form colonies [53]. To assess the in vivo effect of estrogen on *GREB1* expression, Rae et al. ovariectomized athymic nude mice to prevent endogenous estrogen production and implanted MCF-7 cells in the peritoneal cavity [10]. As expected, the levels of *GREB1* and estrogen were reduced upon ovariectomization. Conversely, *GREB1* expression increased when exogenous estrogen was administered. Although estrogen treatment did not affect tumor size during the 48-hour treatment period [10], longer treatment could result in enhanced tumor growth, as suggested by in vitro work showing that estrogen induced the proliferation of MCF-7, BT-474, and T47D cell lines [10]. 

Further evidence for the role of *GREB1* in breast cancer comes from studies of the cells’ responses to Tamoxifen. Breast cancer cells initially respond to Tamoxifen, which antagonizes the ER and leads to decreased *GREB1* expression [65]. However, in Tamoxifen-resistant breast cancer tissues, the *GREB1* promoter becomes more methylated via the action of the histone lysine N-methyltransferase EZH2, leading to the epigenetic downregulation of *GREB1* [65]. 

## 6. Role of GREB1 in Ovarian Cancer

GREB1 also plays a significant role in ovarian cancer, which is a gynecologic malignancy that causes an estimated 12,700 deaths in the United States (US) alone [66]. Unlike breast tumors, where the tissue is easily accessible for diagnostic purposes, the ovary is located deep in the pelvic cavity. Therefore, the early detection of ovarian cancer proves difficult due to the subtle symptomology of cancer and poor screening methods [67]. As a result, nearly 75% of ovarian cancers are diagnosed only in later (advanced) stages of cancer [66,67]. Ovarian cancers are traditionally classified into three main categories with additional subtypes based on their histogenesis and subsequent differentiation [68]. Sex cord stromal tumors develop in the connective tissue of the ovaries and account for approximately 5% of ovarian cancers in women. Similarly, germ cell tumors account for about 5% of ovarian cancers, and are typically diagnosed in young women aged aroundearly 20 years. Accounting for almost 90% of cases, epithelial ovarian cancers are classified into several subtypes, of which serous tumors are the most notable [66]. A key study reported that 86% of serous epithelial ovarian tumors are ER-positive [15]. In vitro, estrogen can stimulate the proliferation of ER-positive ovarian cancer cell lines [69]. Several lines of evidence point to a role for GREB1 in the progress of epithelial ovarian cancer. In a tissue microarray analysis of ovarian cancer cases, GREB1 was expressed in 75–85% of epithelial ovarian cancer cells [55]. Tumor xenograft studies using mouse ovarian cancer ascites (MAS) cells with elevated GREB1 expression indicated an increase in tumor growth and reduced median survival time [16,55]. In vitro, GREB1 knocked down in MAS cells results in decreased proliferation and favorable morphologic changes [16,55]. These findings support a key role for GREB1 in estrogen-dependent actions in epithelial ovarian cancers.

## 7. Role of GREB1 in Prostate Cancer

Accounting for nearly 366,000 deaths globally per year, prostate cancer is the second most prevalent cancer in males in the US, and the majority of cases are adenocarcinomas [70,71]. Similar to the hormonal regulation of reproductive tract development and sexual function by estrogen in women, androgen controls the central reproductive function in males, including the prostate [72]. The prostate is an excretory gland that secretes prostate fluid, and the prostatic glands are androgen-dependent in nature. The initiation and progression of prostate cancer are regulated by androgen [73]. The common treatment options for prostate cancer include orchiectomy and hormonal-deprived therapy, both of which function by restricting androgen/androgen receptor (AR) functions [4]. Androgen can regulate *GREB1* expression by binding to ARs, which then bind the ARE upstream of the *GREB1* transcriptional start site (Figure 2) [44]. Rae et al. showed that GREB1 was highly expressed in benign prostatic hypertrophy, prostate cancer, and AR-positive prostatic carcinoma cell lines, and that GREB1 responded to androgen in a dose-dependent manner. Additionally, they reported that GREB1 knockdown reduced the proliferation of lymph node carcinoma of the prostate (LNCaP) cells [44]. Similarly, Ferreira, et al. found that treatment with the androgen dihydrotestosterone upregulated *GREB1* expression in androgen-responsive LNCaP cells [74]. 

## 8. The Emerging Role for GREB1 in Other Hormone-Dependent Cancers

Although not as well studied as in breast, ovary, and prostate cancers, GREB1 may also play a role in uterine and testicular cancer. The uterus is composed of the endometrium and myometrium. Among women of reproductive age, the cyclical fluctuation of estrogen and progesterone in the endometrium promotes the monthly proliferation, differentiation, and shedding of tissue, which is commonly known as menstruation [72]. In the uterus, *GREB1* is highly expressed in the endometrium [59]. Throughout women’s reproductive age, *GREB1* expression fluctuates in accordance with estrogen levels [59]. Endometrial tissues under the long-term influence of estrogen are disposed to endometrial hyperplasia and endometrial adenocarcinoma. In addition to being regulated by estrogen, recent evidence indicates that *GREB1* is regulated by progesterone in human endometrial cells [40]. *GREB1* expression increased during progesterone-mediated endometrial stromal cell decidualization, and progesterone receptor knockdown led to a decrease in *GREB1* transcript levels [40]. Given that endometrium exposed to estrogen in the absence of progesterone is prone to hyperplasia and carcinogenesis [72] and that GREB1 is regulated by both hormones, it seems likely that GREB1 contributes to endometrial cancer. One study reported a positive correlation between GREB1 expression and ERα activation in endometrial cancer [75]. Additionally, endometrial cancer appears to overlap genetically with endometriosis [76,77], and *GREB1* mRNA levels are significantly higher in ectopic endometrium (endometriosis lesions) than in eutopic endometrium [78]. Furthermore, single nucleotide polymorphisms around the *GREB1* locus have been identified in endometriosis patients [59,79,80,81]. Further, *GREB1* polymorphic variants are associated with bone mineral density in a cohort of Caucasians [82]. Low bone mineral density is a critical manifestation of osteoporosis, and estrogen is known to control bone remodeling. Thus, future work should address the possibility that mutations in *GREB1* predispose women to hormone-related diseases including endometriosis and postmenopausal osteoporosis.

GREB1 may also participate in uterine sarcomas, which constitute 8% of uterine corpus cancers [83] and are derived from myometrial, mesenchymal, or undifferentiated cells [84]. Brunetti et al. reported a translocation (2;8)(p25;q13) in a patient with undifferentiated uterine sarcoma and lung metastases. Transcriptome sequencing analysis revealed that this translocation created a novel *GREB1–nuclear receptor coactivator 2 (NCOA2)* fusion gene in which the first three exons of *GREB1* (NM_014668.3) were fused to exon 15 of *NCOA2* (NM_001321703.1) [41]. Given that *NCOA2*, which is also called *SRC-2*, regulates the progesterone-dependent transcriptional regulation of GREB1 in human endometrial cells [40], additional studies are required to determine the role of this fusion gene in endometrial cancer onset and progression.

The testes, which both produces gametes and secretes androgen [72] to maintain sexual characteristics [85], relies on androgen signaling for many of its functions. No work has been done to assess the role of GREB1 in testicular cancer, but some findings suggest that GREB1 participates in testicular functions [86,87]. The Sertoli cells, which establish the blood–testis barrier in the seminiferous tubules, express both androgen and estrogen receptors [88]. Two papers suggested that *GREB1* is expressed in mouse Sertoli cells as a result of estrogen binding to the ER [86,87]. Given these findings, future work should be directed at assessing whether or not GREB1 participates in testicular functions and testicular cancer.

## 9. Translational Relevance

Understanding the underlying mechanism of hormone actions will offer novel diagnostic and/or treatment strategies in the clinical management of hormone-dependent cancers. Considering the role of GREB1 in hormone-dependent cancers, GREB1 should be considered as a diagnostic and therapeutic target. In breast cancer, Mohammed et al. found that the level of GREB1 was a favorable prognostic factor independent of the Nottingham prognostic index, which predicts breast cancer prognosis after surgery on the basis of lesion size, the number of involved lymph nodes, and tumor grade [53,89]. Similarly, in ovarian cancer, the hypomethylation of *GREB1* was positively correlated with progression-free survival in ovarian cancer patients [90]. Additionally, a microarray analysis of prostate cancer samples from 33 patients showed that high *GREB1* expression was associated with organ-confined disease, which is lower stage and a more favorable prognosis in prostate cancer [91]. It important to note that elevated expressions of GREB1 have been observed in hormone-dependent cancers, which are more sensitive to hormone therapy than receptor-negative tumors. Therefore, GREB1 expression in lower stage receptor-positive cancers might be more favorable toward prognosis. The majority of higher stage tumors from the aforementioned report [91] might be receptor-negative, which are resistant to therapy, and thus are not favorable for prognosis. Nonetheless, future work should conduct similar studies in larger cohorts of patients from multiple hormone-dependent cancers.

In addition to informing prognosis, the improved understanding of GREB1 may reveal novel strategies to combat drug resistance. In Tamoxifen-resistant breast cancer tissues, the *GREB1* promoter becomes more methylated via the action of the histone lysine N-methyltransferase EZH2, leading to the epigenetic downregulation of *GREB1* [65]. EZH2-mediated histone methylation drives the epigenetic programming in tumor cells, including breast, ovarian, and prostate cancers [92,93,94,95]. These epigenetic changes activate multiple oncogenes and promote tumor progression and metastasis [96]. Thus, it is plausible that *GREB1* methylation confers drug resistance for hormone-dependent cancers. Based on the aforementioned studies, strategies to target GREB1 could lead to improved hormone-dependent cancer prognosis and treatment.

Interestingly, the expression analysis of GREB1 demonstrated in Oncomine, the Cancer Cell Line Encyclopedia (CCLE), and protein atlas indicate that GREB1 has much wider expression than in hormone-related organs or tissues. Generally, the expression level of GREB1 is much higher in pathologic status than in healthy status. Thus, a small molecular inhibitor or monoclonal antibody against GREB1 that abolishes the overexpressed level of GREB1 with minimal effect on physiological actions would be a viable option to target GREB1. Thus, an in-depth functional analysis of GREB1 in hormone-dependent cancers is warranted prior to targeting GREB1 in hormone-dependent cancers.

## 10. Conclusions and Open Questions

The pan hormone-responsive gene GREB1 plays important roles in the initiation and progression of some sex hormone-driven cancers. However, many open questions remain, such as: (1) Do GREB1 isoforms have differential actions in hormone-dependent cancers? If so, what are the underlying mechanisms? (2) Is GREB1 epigenetically regulated in cancer? (3) Does GREB1 function via common or hormone-specific functions? (4) Are mutations in GREB1 associated with the occurrence of hormone-dependent cancers? By addressing such questions, the field will be able to fully elucidate the normal functions of GREB1, define its role in cancers, and assess its potential as a prognostic and therapeutic target.

## Figures and Tables

**Table 1:** The Protein Expression of GREB1 and Hormone Receptor in Different Tissue Types. ER: Estrogen Receptor, AR: Androgen Receptor, PR: Progesterone Receptor.

Tissue Type and Expression	GREB1		ER		AR		PR	
	Normal	Cancer	Normal	Cancer	Normal	Cancer	Normal	Cancer
Breast	+	+	ER-α +	ER-α +	+	+	+	+
			ER-β −	ER-β +				
Ovary	+	+	ER-α −	ER-α +	+	+	−	+
			ER-β −	ER-β +				
Endometrium	+	+	ER-α +	ER-α +	+	+	+	+
			ER-β −	ER-β +				
Myometrium of Uterus	+	?	ER-α +	ER-α ?	+	?	+	?
			ER-β −	ER-β ?				
Prostate	+	+	ER-α −	ER-α +	−	+	−	+
			ER-β −	ER-β +				
Testis	+	+	ER-α −	ER-α +	+	+	−	+
			ER-β +	ER-β +				

+: expression; −: no expression; ?: not mentioned. (The expression of tissue is according to the data shown on the website of human protein atlas: https://www.proteinatlas.org/).

## Abbreviations

AD	Activation Domain
AR	Androgen Receptors
ARE	Androgen Response Element
b-HLH-PAS	Basic Helix-Loop-Helix-Per/ARNT/Sim
CCLE	Cancer Cell Line Encyclopedia
ChIP	Chromatin Immunoprecipitation
ER	Estrogen Receptors
ERE	Estrogen Response Element
GREB1	Growth Regulation by Estrogen in Breast Cancer 1
HR	Hormone Receptors
HRE	Hormone Response Element
LBD	Ligand Binding Domain
LNCaP cell	Lymph Node Carcinoma of the Prostate cell
LXXLL	Leu-X-X-Leu-Leu
MAS cell	Mouse Ovarian Cancer Ascites Cell
NCOA2	Nuclear Receptor Coactivator 2
PR	Progesterone Receptors
SERM	Selective Estrogen Receptor Modulator
SF II	Superfamily II
SRC	Steroid Receptor Coactivator
TAGT	TET/JBP-Associated Glycosyltransferase
TET/JBP	Ten-Eleven Translocation/J Binding Protein
